# Tumour-stroma ratio outperforms tumour budding as biomarker in colon cancer: a cohort study

**DOI:** 10.1007/s00384-021-04023-4

**Published:** 2021-09-17

**Authors:** Marloes A. Smit, Gabi W. van Pelt, Valeska Terpstra, Hein Putter, Rob A. E. M. Tollenaar, Wilma E. Mesker, J. Han J. M. van Krieken

**Affiliations:** 1 Department of Surgery, Leiden University Medical Center, Leiden, The Netherlands; 2 Department of Pathology, Haaglanden Medical Center, The Hague, The Netherlands; 3 Department of Medical Statistics, Leiden University Medical Center, Leiden, The Netherlands; 4 Department of Pathology, Radboud University Medical Center, P.O. Box 9101, 6500 HB Nijmegen, The Netherlands

**Keywords:** Colon cancer, Tumour-stroma ratio, Tumour budding, Tumour microenvironment, Personalised medicine

## Abstract

The tumour-stroma ratio (TSR) and tumour budding (TB) are two high-risk factors with potential to be implemented in the next TNM classification. The aim of the current study was to evaluate the practical application of the two biomarkers based on reproducibility, independency and prognostic value. Patients diagnosed with stage II or III colon cancer who underwent surgery between 2005 and 2016 were included. Both TSR and TB were scored on haematoxylin and eosin-stained tissue sections. The TSR, based on the relative amount of stroma, was scored in increments of 10%. TB was scored following the consensus guidelines; a bud was defined as ≤ 4 tumour cells. For analysis, three categories were used. Cohen’s kappa was used for reproducibility. The prognostic value was determined with survival analysis. In total, 246 patients were included. The TSR distribution was *N* = 137 (56%) stroma-low and *N* = 109 (44%) stroma-high. The TB distribution was TB-low *N* = 194 (79%), TB-intermediate *N* = 35 (14%) and TB-high *N* = 17 (7%). The reproducibility of the TSR was good (interobserver agreement kappa = 0.83 and intraobserver agreement kappa = 0.82), whereas the inter- and intraobserver agreement for scoring TB was moderate (kappa 0.47 and 0.45, respectively). The survival analysis showed an independent prognostic value for disease-free survival for TSR (HR 1.57; 95% CI 1.01–2.44; *p* = 0.048) and for TB-high (HR 2.01; 95% CI 1.02–3.96; *p* = 0.043). Based on current results, we suggest the TSR is a more reliable parameter in daily practice due to better reproducibility and independent prognostic value for disease-free survival.

**Supplementary Information:**

The online version contains supplementary material available at 10.1007/s00384-021-04023-4.

## Introduction

The prognosis and selection for adjuvant treatment of colon cancer patients is largely based on the Tumour Node Metastasis (TNM) classification [3]. Patients diagnosed with stage III or stage II with one or more high-risk (ASCO) criteria will usually be selected for adjuvant chemotherapy [3]. However, among patients staged II without any high-risk factors, approximately 30% will suffer from recurrent disease within 3 to 5 years after surgery [28]. To better predict which patients will develop recurrence, additional high-risk factors next to the ASCO criteria have been described [3]. These “new” high-risk factors should improve the selection of patients who will likely benefit from adjuvant therapy. Thus, high-risk criteria should not only select stage II patients at high risk for recurrence, but also select patients at stage III who are likely to be overtreated with adjuvant therapy.

New prognostic parameters have been identified not only on the basis of molecular pathology (for example CMS analysis) [13] and lymph node assessment (for example one-step nucleic acid amplification assay (OSNA)) [1, 5], but also on simple morphologic parameters. Morphologic parameters are tissue based and can be evaluated during routine pathology practice.

The tumour-stroma ratio (TSR) is a biomarker based on the microenvironment of the tumour and has proven to be a strong prognostic parameter [29, 30]. The TSR is based on the relative amount of stroma in the primary tumour. Patients with a tumour containing > 50% stroma (stroma-high) have a worse prognosis, compared to patients with a tumour of ≤ 50% stroma (stroma-low). The TSR is validated by many international study groups and is prognostic in multiple epithelial cancer types [29, 30]. The TSR is scored on haematoxylin and eosin (H&E)-stained sections used in routine diagnostics; the scoring method is easy to learn and well reproducible and takes about 1–2 min [27].

Tumour budding (TB), the propensity of the primary tumour to bud off single cells and cell clusters (≤ 4 cells) at the invasive front, correlates with prognosis and is also frequently evaluated as a new biomarker in colon cancer. According to the guidelines, TB scoring should be performed at the invasive front of a tumour on an H&E-stained section [20]. The reproducibility of TB on H&E sections shows highly variable results [7, 12, 17, 18]. Therefore, some studies use cytokeratin-stained sections to identify the tumour buds for better interobserver agreement [10, 16]. Various studies showed TB to be an independent prognostic biomarker for overall survival (OS) and disease-free survival (DFS) in stage I and stage II colon cancer patients [9, 16, 24]. Patients with tumours with high budding have a worse prognosis compared to patients with low budding. Recently, it was recommended to report TB in T1 tumours for decision-making about additional resection after biopsy or removal of a polyp [2].

Both TSR and TB have shown to be prognostic biomarkers in several series of colon cancer patients and both seem potentially suitable to use in routine pathology diagnostics. In order to implement TSR and/or TB as prognostic factors in daily clinical practice, their robustness and reproducibility should be thoroughly assessed [4]. TB has recently been added to the guidelines for locally advanced colon cancer [2]. The prospective validation of the TSR as a biomarker is currently under investigation in the UNITED study [25, 26].

Here, we analyse the value of TSR and TB by comparing their reproducibility, independency from one another and the prognostic value in stage II and stage III colon cancer patient samples.

## Materials and methods

### Patient selection

Patients who underwent curative surgery for colon cancer, between January 2005 up to and including December 2016 at the LUMC, were retrospectively included in this cohort study. Patients were included when they met the following inclusion criteria: pathological stage II or stage III colon cancer and age ≥ 18 years. The following exclusion criteria were met: rectal cancer, neo-adjuvant treatment, a medical history of cancer 10 years prior to colon cancer (except for basal cell skin cancer or cervical carcinoma in situ) or any colon cancer in history, double tumours, and/or deceased within 3 months after surgery (Supplementary table 1). The H&E-stained slides used for routine diagnostics were collected from the Department of Pathology and the slides were anonymised and scanned with the Panoramic 250 scanner (3DHistech, Hungary) (tissue level pixel size ~ 0.33 µm/pixel) for digital analysis. The observers were blinded for clinical and pathological data and for each other’s results during biomarker scoring.

### Tumour-stroma ratio

**Fig. 1:**
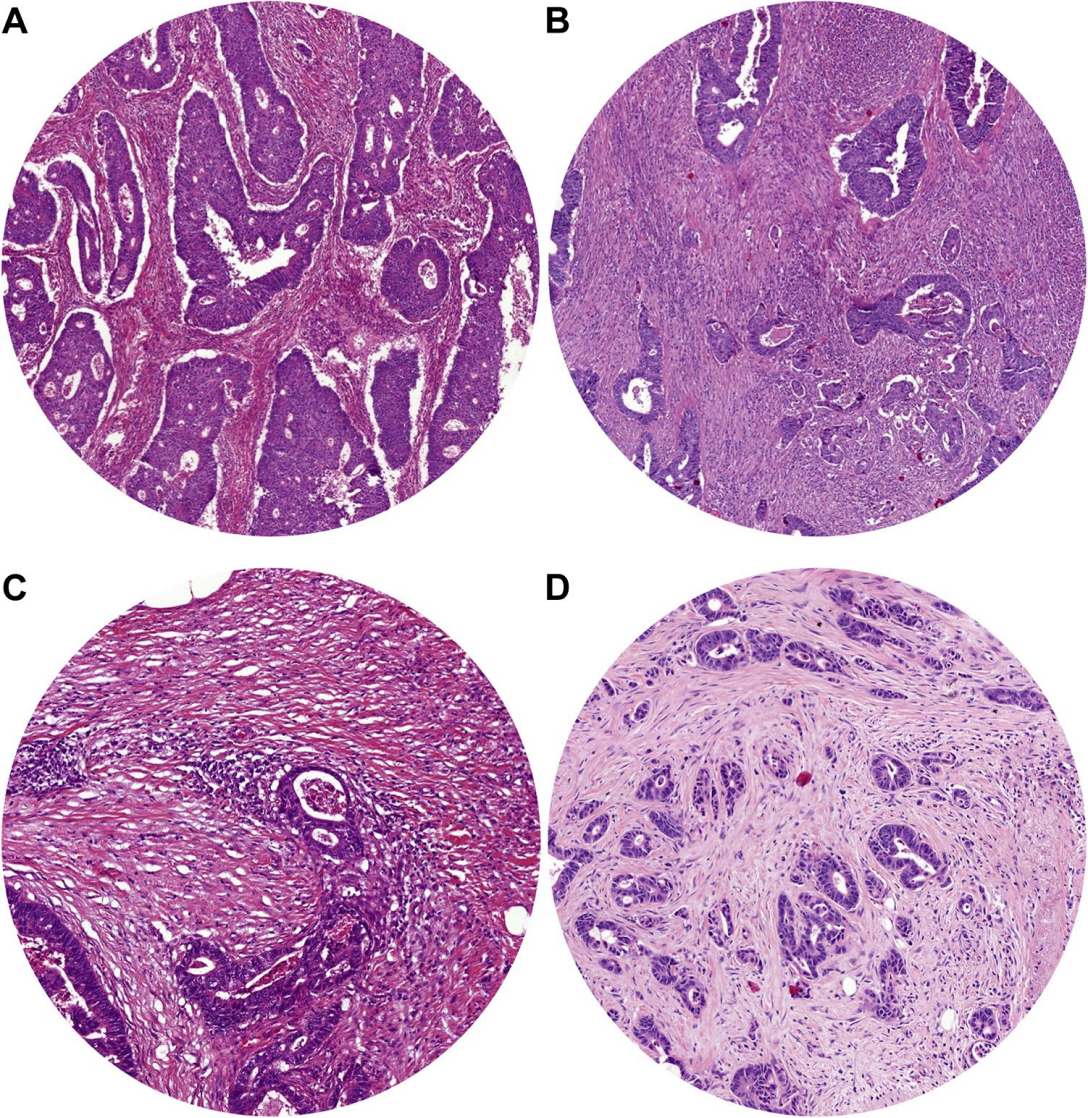
Examples of the 4-μm haematoxylin and eosin-stained slides of colon carcinomas. In **A**, a stroma-low tumour; in **B**, a stroma-high tumour. Both viewed at a 100 × magnification with an area of 3.4mm^2^. In **C**, a tumour-budding low tumour; in **D**, a tumour-budding high tumour. Scored in an area of 0.785mm^2^

The TSR was scored on H&E-stained sections from the primary tumour by two observers (MS and GvP, Leiden University Medical Center, Leiden). The TSR was scored at a 100 × magnification. The stroma percentage was scored in increments of 10, in a field with as much as possible tumour-stroma and with tumour cells on four opposite sides of the vision field [22, 23, 27]. If no agreement was reached, a third observer was consulted (HvK, Radboud University Medical Center, Nijmegen). One of the observers (MS) scored the TSR also digitally, using a circular annotation of 3.4mm^2^ to mimic the field of view of a 100 × magnification. For analysis, the TSR was dichotomised. A tumour with an amount of stroma of ≤ 50% was classified as stroma-low, and a percentage > 50% was classified as stroma-high, in line with previous studies [15, 22, 23, 27]. In Fig. 1, an example of a stroma-low (A) and a stroma-high tumour (B) is shown.

### Tumour budding

TB was scored, on exactly the same slides as the TSR, by two observers (VT (Haaglanden Medical Center, the Hague) and HvK) as recommended by the consensus [20]. HvK scored TB both microscopically and digitally, and VT scored TB only digitally. A tumour bud was determined as a single cell or a small cluster of cells to a maximum of four cells. TB was scored at the invasive front, at a single vision field by a magnification of 200 × . The number of buds was normalised as described in the conversion table in the consensus. When TB was scored digitally, an annotation with an area of 0.785mm^2^ was used. For survival analysis, the microscopic numbers were used, and the continuous numbers were categorised for statistical analysis. The three categories were TB-low (0–4 buds), TB-intermediate (5–9 buds) and TB-high (≥ 10 buds) [20]. In Fig. 1, an example of a TB-low (C) and a TB-high tumour (D) is shown.

### Statistics

Descriptive variables are presented with mean and standard deviation (SD) for normally distributed continuous variables. Non-normally distributed continuous variables are presented by median and range. The chi-square test is used for measuring associations between categorical variables. Cohen’s kappa is used to determine the interobserver agreement of scoring TSR and TB (digitally) and to determine the intraobserver agreement for scoring TSR and TB (microscopic vs digital).

The prognostic value of the two individual parameters was explored. DFS was defined as the time from surgery to recurrence or death, depending on what occurred first. OS was defined as the time from surgery to death of any cause.

Univariate survival analysis was performed using a Kaplan-Meijer curve and a log rank test. Cox regression analysis was performed for univariate and multivariate analysis for hazard ratios (HR) and the 95% confidence interval (95% CI).

All tests were 2-sided and a *p*-value of < 0.05 was considered to be significant. Statistical analyses were performed using SPSS version 25 (SPSS Inc., Chicago, IL, USA).

## Results

### Patient cohort

**Fig. 2:**
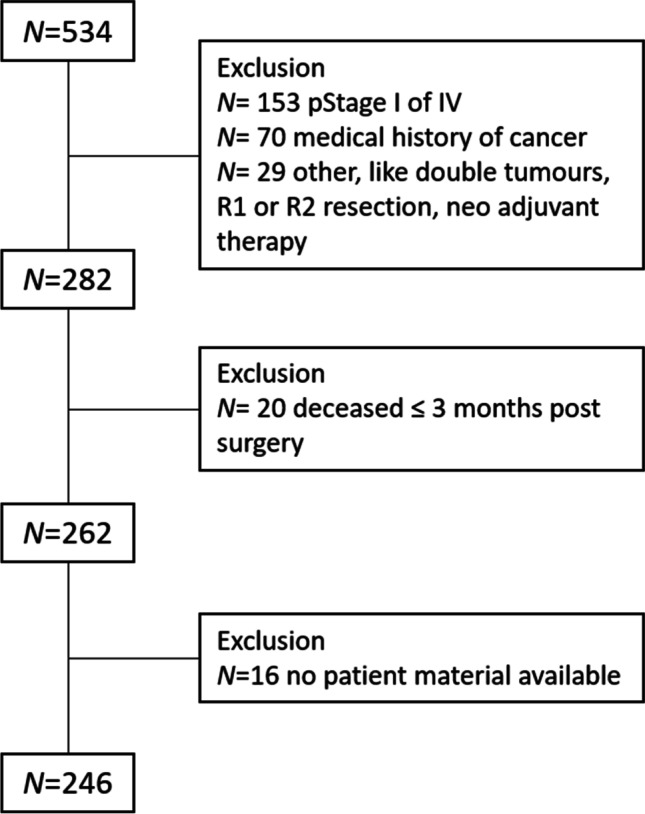
Flowchart of the patient selection

In total, 381 colon cancer stage II or stage III patients underwent surgery in the time period 2005–2016. Of these, 135 patients were excluded because one of the exclusion criteria was met, most often (*N* = 70) due to a medical history of cancer, and 246 patients were included in the cohort (Fig. 2). The tumours of these patients were scored for both TSR and TB.

The patient population in the cohort had a mean age of 68 years (SD 12 years) and 54% males (*N* = 134). Fifty-three per cent (*N* = 131) of the patients had pathological stage (p-Stage) II and 92 patients (37%) received adjuvant therapy. The TSR distribution was *N* = 137 (56%) stroma-low and *N* = 109 (44%) stroma-high. TB was divided in 3 categories: TB-low (*N* = 194 (79%)), TB-intermediate (*N* = 35 (14%)) and TB-high (*N* = 17 (7%)). All baseline characteristics are displayed in Table 1.

**Table 1:** Patient and tumour characteristics of 246 patients with colon cancer

Characteristics	All	Tumour-stroma ratio			Tumour budding			
		Stroma-low (≤ 50%)	Stroma-high (> 50%)		Low (0–4 buds)	Intermediate (5–9 buds)	High (≥ 10 buds)	
	*N* = 246 (%)	137 (56)	109 (44)	*p*-value	194 (79)	35 (14)	17 (7)	*p*-value
Age (years), mean (SD)	68 (12)	68 (12)	67 (12)	0.349	68 (12)	65 (11)	69 (13)	0.282
Gender				0.166				0.456
Male	134 (54)	80 (58)	54 (50)		109 (56)	18 (51)	7 (41)	
Female	112 (46)	57 (42)	55 (51)		85 (44)	17 (49)	10 (59)	
pTNM stage				0.298				0.987
II	131 (53)	77 (56)	54 (50)		104 (54)	18 (51)	9 (53)	
III	115 (47)	60 (44)	55 (51)		90 (46)	17 (49)	8 (47)	
pT status				0.566				0.506
T1 + T2	18 (7)	17 (12)	54 (50)		17 (9)	1 (3)	0 (0)	
T3	196 (80)	106 (78)	36 (33)		152 (78)	30 (86)	14 (82)	
T4	32 (13)	14 (10)	19 (17)		25 (13)	4 (11)	3 (18)	
pN status				0.566				0.986
N0	131 (53)	77 (56)	54 (50)		104 (54)	18 (51)	9 (53)	
N1	74 (30)	38 (28)	36 (33)		59 (30)	10 (29)	5 (29)	
N2	41 (17)	22 (16)	19 (17)		31 (16)	7 (20)	3 (18)	
Localisation				0.002*				0.859
Right	117 (48)	77 (56)	40 (37)		93 (48)	17 (49)	7 (41)	
Left	129 (52)	60 (44)	69 (63)		101 (52)	18 (51)	10 (59)	
Adjuvant therapy				0.553				0.106
No	154 (63)	88 (64)	66 (61)		124 (64)	17 (49)	13 (77)	
Yes	92 (37)	49 (36)	43 (39)		70 (36)	18 (51)	4 (24)	

^*^Significant result

*N* lymph nodes,* p* pathological, *SD* standard deviation, *T* tumour

### Interobserver variability

The interobserver agreement for scoring TSR between the two observers was good to almost perfect (kappa = 0.83). The TSR was also scored digitally by one observer (MS), and a good to almost perfect intraobserver agreement was reached (kappa = 0.82).

The interobserver agreement for scoring TB was moderate with a kappa of 0.47. One of the observers (HvK) scored the sections microscopically and digitally for TB, with a moderate intraobserver agreement of kappa 0.45. A wide variety of scoring was observed when reviewing the discrepancies, even within one case, and no trends or obvious reason for discrepancy could be detected that could explain the inter- or intraobserver variation.

### Association

Of the 246 patients, 120 (49%) were categorised as stroma-low and TB-low (low-risk patients), and 10 (4%) patients were classified as stroma-high and TB-high (high-risk patients). The distribution of TSR and TB is shown in supplementary Table 2. An association between TSR and TB was found (chi-square *p* = 0.001).

### Survival analysis

The median follow-up time was 47 months (range 4–158). During follow-up, 48 (20%) patients had recurrence of disease, and 68 (28%) patients died. In total, 83 (34%) DFS events occurred, due to the fact that some patients deceased with recurrence.

**Fig. 3:**
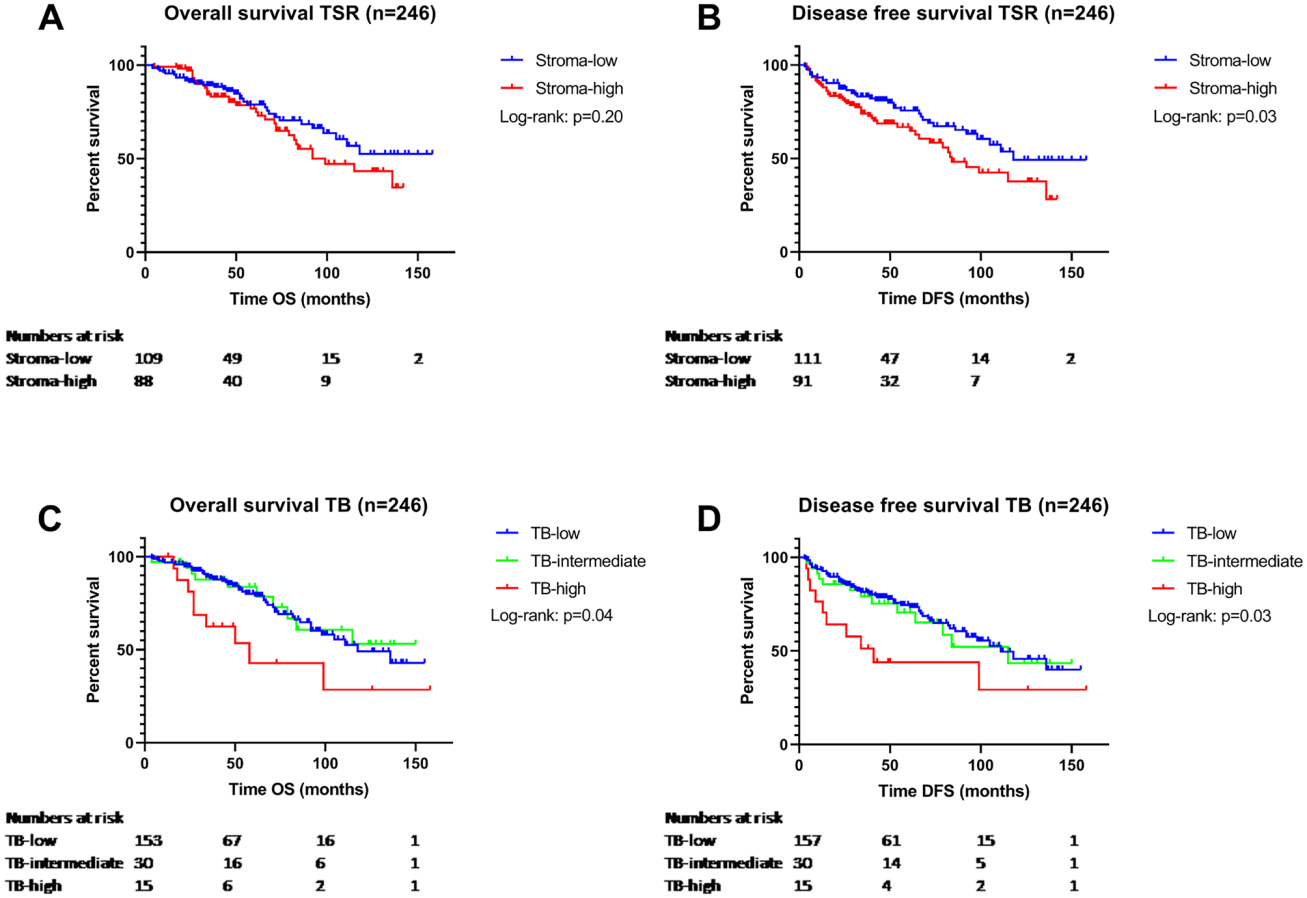
The Kaplan-Meijer survival curves of the 246 patients with colon cancer. Survival curves for TSR in **A** for overall survival (*p* = 0.20) and **B** for disease-free survival (log rank *p* = 0.03). Survival curves for TB in **C** for overall survival (*p* = 0.04) and in **D** for disease-free survival (*p* = 0.03)

There was no significant difference in OS for TSR (HR 1.36; 95% CI 0.84–2.19; *p* = 0.206). However, the TSR was prognostic for DFS (HR 1.59; 95% CI 1.03–2.45; *p* = 0.036). Univariate analysis showed that TB was prognostic for OS (TB-high HR 2.36; 95% CI 1.16–4.81; *p* = 0.018) and for DFS (TB-high HR 2.40; 95% CI 1.23–4.70; *p* = 0.011). Kaplan-Meijer survival curves for TSR and TB are shown in Fig. 3.

Based on the results from the univariate Cox regression analysis (Table 2), in the multivariate Cox regression model, the results were corrected for age and pT-status. TSR remained a significant prognostic parameter for DFS (HR 1.57; 95% CI 1.01–2.44; *p* = 0.048), but this prognostic value was not found for OS. For TB, the prognostic value did not retain/remain significant in multivariate analysis for OS, but for DFS, TB-high remained prognostic (HR 2.01; 95% CI 1.02–3.96; *p* = 0.043) (Table 3).

**Table 2:** Cox univariate analysis for overall and disease-free survival

		Overall survival			Disease-free survival		
	*N* (%)	HR	95% CI	*p*-value	HR	95% CI	*p*-value
Age	246 (100)	1.069	1.041–1.097	< 0.001	1.052	1.029–1.076	< 0.001
Gender							
Male	134 (54)	REF		0.888	REF		0.418
Female	112 (46)	0.966	0.597–1.563		1.196	0.776–1.842	
pTNM stage							
II	131 (53)	REF		0.621	REF		0.369
III	115 (47)	1.128	0.700–1.817		1.219	0.792–1.875	
pT status							
T1 + T2	18 (7)	REF		0.191	REF		0.051
T3	196 (80)	1.271	0.396–4.841	0.686	1.813	0.569–5.777	0.315
T4	32 (13)	2.138	0.613–7.460	0.233	3.276	0.954–11.256	0.059
pN status							
N0	131 (53)	REF		0.687	REF		0.604
N1	74 (30)	1.034	0.598–1.786	0.906	1.161	0.711–1.894	0.551
N2	41 (17)	1.330	0.691–2.560	0.393	1.334	0.739–2.408	0.339
Localisation							
Right	117 (48)	REF		0.955	REF		0.127
Left	129 (52)	0.986	0.612–1.590		0.946	0.434–1.10	
Adjuvant therapy							
No	154 (63)	REF		0.005*	REF		0.127
Yes	92 (37)	0.441	0.249–0.783		0.694	0.434–1.110	
TSR							
Stroma-low	137 (56)	REF		0.206	REF		0.036*
Stroma-high	109 (44)	1.359	0.844–2.188		1.589	1.032–2.447	
TB							
Low	194 (79)	REF		0.051	REF		0.039
Intermediate	35 (14)	0.924	0.467–1.828	0.820	1.116	0.612–2.035	0.721
High	17 (7)	2.358	1.157–4.805	0.018*	2.397	1.225–4.693	0.011*

^*^Significant result

*95% CI* 95% confidence interval,* N* Lymph nodes,* p* pathological, *SD* standard deviation, *T* tumour, *TB* tumour budding, *TSR* tumour-stroma ratio

**Table 3:** Cox multivariate analysis for overall and disease-free survival, corrected for age and pT-status

		Overall survival			Disease-free survival		
	*N* (%)	HR	95% CI	*p*-value	HR	95% CI	*p*-value
TSR							
Stroma-low	137 (56)	REF		0.151	REF		0.048*
Stroma-high	109 (44)	1.432	0.877–2.338		1.565	1.005–2.437	
TB							
Low	194 (79)	REF		0.144	REF		0.103
Intermediate	35 (14)	1.21	0.601–2.442	0.592	1.358	0.736–2.505	0.328
High	17 (7)	2.069	1.000–4.283	0.050	2.013	1.022–3.964	0.043*

^*^Significant result

## Discussion

In the current study, two morphology-based histological parameters were evaluated and correlated with the prognosis of stage II and stage III colon cancer patients. Both parameters are easy to assess in daily routine pathology, as they are scored on H&E-stained sections. This study showed that TSR was an independent prognostic parameter for DFS, but not for OS. TB was a prognostic parameter for OS as well as for DFS in the univariate analysis, but did not remain significant as an independent prognostic parameter after multivariate analysis. No clear explanation could be found why the OS for TSR was not significantly different between the stroma-low and stroma-high group. When observing the survival curves, in the first year after surgery, more people died in the stroma-low group. At baseline, the stroma-low group was slightly older and more often at stage III; however, these groups were not significantly different. Elderly patients are generally at higher risk for developing late surgery-related complications and may die due to these complications [8, 11]. TB was probably not prognostic due to the fact that the group TB-high was small (*N* = 17 (7%)). However, TB is recommended by the ESMO guidelines for localised colon cancer to score in daily diagnostics [2]. The prognostic significance of TB was evaluated by Landau et al. in a cohort of stage III colon cancer patients, showing TB to be an independent prognostic parameter for recurrence-free survival [19]. In contrast, analysing the prognostic effect of TB in all stages of colon cancer TB failed to be significant as an independent prognostic factor, except when stage II patients were analysed separately [6]. In the current study, we did not analyse stage II and stage III separately, due to the low number of patients with TB-high score (stage II 10 patients, stage III 7 patients).

The TSR and TB were both scored by two observers, as is preferred in the research setting. The interobserver agreement of scoring TSR was good to almost perfect (kappa = 0.83), and this result is comparable with current literature [27]. The interobserver agreement for scoring TB has shown to be moderate (kappa = 0.47), as was the intraobserver agreement (kappa = 0.45). The interobserver variability for TB is diverse [7, 12, 17, 18], and our results are consistent with previous research [14, 21].

In daily pathology practice, there is currently a shift towards digital microscopy. Therefore, we compared the microscopical and digital scoring for both TSR and TB. The TSR was well reproducible (intraobserver agreement of kappa = 0.82). TB however showed only a moderate agreement between the microscopical assessment and the digitalised image assessment (intraobserver agreement of kappa = 0.45).

It is remarkable that TB-low and TB-intermediate show similar overall survival curves. Only TB-high showed a significant worse prognosis compared to the other two groups. In our study, the TB-high group is small with 7% (*N* = 17) of the cases in this group, which is comparable with the findings of Eriksen et al. [10]. In their study, TB was scored on cytokeratin-stained sections and the score was divided into two groups with the cut-off point at 10 buds (≥ 10 buds = budding-high). Here, TB was not significant for survival. We may conclude that TB-high is a prognostic factor, but only for a small subgroup of the patient population. Eriksen et al. also investigated the prognostic value of the TSR and showed that the TSR was independently prognostic for survival (DFS and OS).

An association between TSR and TB was found. The hypothesis is that patients who are stroma-high and TB-high have a significant worse survival compared to stroma-low and TB-low patients. It would be interesting to investigate this combined parameter for impact on survival, but the patient groups in our study were too small to draw reliable conclusions.

As all retrospective cohort studies, this design is a limitation of the current study. As a benefit of the retrospective design, long-time follow-up data was available for all patients in the cohort. The number of patients in the cohort should preferentially be larger and needs validation in an independent validation cohort. The UNITED study, a multicentre prospective study, could serve as a good potential [25].

Both TB and TSR are scored on H&E-stained sections and can thus be scored during routine diagnostics. Comparing both methods, TSR is a fast and easy parameter to score and is highly reproducible compared to TB. Some pathologists prefer to score TB after the slide is stained for cytokeratin for better visualisation of the tumour buds. This certainly helps to increase the reproducibility, but also makes the scoring more costly and time consuming.

Regarding the simplicity and consistency of assessing TSR and its independent prognostic value for disease-free survival of stage II and III colon cancer patients, we suggest that adding TSR as a biomarker in the pathology report could be of value in clinical decision policy.

## Supplementary Information

Below is the link to the electronic supplementary material.

Supplementary file1 (PDF 144 kb)

Supplementary file2 (PDF 134 kb)

## Data Availability

The dataset analysed during the current study is available from the corresponding author on reasonable request.
