# Supplementary material for: Tumour-stroma ratio outperforms tumour budding as biomarker in colon cancer: a cohort study
Source: Int J Colorectal Dis. 2021 Sep 17;36(12):2729–37. doi: 10.1007/s00384-021-04023-4 (PMC8589816; doi:10.1007/s00384-021-04023-4)
Supplement: Supplementary file 1 — Supplementary file1 (PDF 144 kb) [file 384_2021_4023_MOESM1_ESM.pdf]

Marloes A Smit, et al. Corresponding author J. Han. JM van Krieken Han.vanKrieken@radboudumc.nl

Supplementary table 1: In- and exclusion criteria

Inclusion criteria	Exclusion criteria
pStage II and III	Age <18
H&E slides available	Rectal cancer
	Neoadjuvant treatment
	Medical history of cancer 10 years prior to colon cancer or any colon cancer in history
	Double tumours
	Deceased within 30 days after surgery
