# Supplementary material for: Tumour-stroma ratio outperforms tumour budding as biomarker in colon cancer: a cohort study
Source: Int J Colorectal Dis. 2021 Sep 17;36(12):2729–37. doi: 10.1007/s00384-021-04023-4 (PMC8589816; doi:10.1007/s00384-021-04023-4)
Supplement: Supplementary file 2 — Supplementary file2 (PDF 134 kb) [file 384_2021_4023_MOESM2_ESM.pdf]

Marloes A Smit, et al. Corresponding author J. Han. JM van Krieken Han.vanKrieken@radboudumc.nl

Supplementary table 2: The association between tumour-stroma ratio and tumour budding. (Chi-square p=0.001)

		Tumour-stroma ratio		
		Stroma-low	Stroma-high	Total
Tumour budding	Low	120 (88%)	74 (68%)	194
	Intermediate	10 (7%)	25 (23%)	35
	High	7 (5%)	10 (9%)	17
Total		137	109	246
